# Quantum-enhanced deep learning models for automated defect detection in materials

**DOI:** 10.3389/frai.2026.1872978

**Published:** 2026-08-20

**Authors:** Aniket Garg, Snehal Sushibine, Diya Choudhuri, Shrey Anand, Helen Vijitha P., Jai Vinita L., Parvathy A. K., Vinaytosh Mishra, Thompson Stephan

**Affiliations:** 1 School of Computer Science and Engineering, Vellore Institute of Technology, Chennai, Tamilnadu, India; 2 Thumbay College of Management and AI in Healthcare, Gulf Medical University, Ajman, United Arab Emirates

**Keywords:** automated material defect detection, computer vision, convolutional neural networks (CNNs), deep learning, hybrid quantum-classical models, quantum-enhanced deep learning, variational quantum circuits (VQCs)

## Abstract

The quality of raw materials is fundamental to the reliability and overall performance of final products, serving as the cornerstone of modern manufacturing standards. While traditional inspection methods can be effective, they are frequently time-consuming, labor-intensive, and unsuitable for high-throughput production environments. Recent advances in quantum computing offer significant advantages for processing large-scale data and complex feature representations, with strong potential to address the limitations of traditional inspection techniques. This study introduces an automated approach for grading raw materials, such as steel and fabric, which are widely used in industries including automotive, textiles, and general manufacturing, by integrating deep learning with quantum machine learning. A Hybrid Quantum Classical Neural Network (HQCNN) is proposed to enhance defect detection. The HQCNN utilizes a fine-tuned ResNet-18 backbone for robust feature extraction. Extracted features are used by a trainable depth controller that determines the complexity of a six-qubit variational quantum circuit (VQC) with data-adaptive, dynamic depth. Each VQC layer consists of rotations around the *Y*-axis (RY) and *Z*-axis (RZ), combined with Controlled-NOT (CNOT) gates arranged in a ring topology, with the total number of layers determined on a per-sample basis. The model is trained using a resource-aware loss function that penalizes excessive circuit depth, and the resulting quantum features are fused with classical features for final classification. Comprehensive ablation studies comparing the HQCNN against parameter-matched classical multi-layer perceptrons (MLPs) and fixed-depth quantum circuits demonstrate the fundamental advantage of the proposed approach. The dynamic HQCNN achieves 88.1 ± 2.4% accuracy on the complex metal surface defect dataset and 93.0 ± 1.8% on the fabric defects dataset. More importantly, it yields a statistically significant improvement in the macro F1-score (from 0.79 in classical parameter-matched baselines to 0.87), proving its superior capability in recognizing complex, minority-class defects while utilizing a fraction of the trainable parameters in the classification head compared to traditional architectures.

## 1 Introduction

In the current manufacturing industry, the increasing need for materials like fabric and steel has led to the requirement for high-quality production. Even small imper- fections in these materials can greatly impact the performance of the product as well as customer satisfaction. Nevertheless, the current manual inspection process still acts as a bottleneck in this industry, as it is time-consuming and prone to human error, which cannot be achieved on a large scale in today's production processes. Recently, deep learning, computer vision, and other developments in the field of Artificial Intel- ligence have significantly improved automated quality control. convolutional neural networks (CNNs) tend to perform better than manual inspection in a controlled envi- ronment in the detection and recognition of non-trivial inconsistencies as they learn to compare hierarchical visual representations of images directly. Methods like low- rank representation (LRR) also enable us to denoise irregularities that are hidden in the irregular texture of material. Nevertheless, the absence of large datasets and the question of whether fully automated solutions are feasible has hindered progress. However, classical deep learning may face challenges in identifying higher-order patterns or quantum correlations in complex materials. To address these challenges, we propose a Hybrid Quantum Classical Neural Network (HQCNN) architecture for automatic defect detection in fabric and steel materials. By integrating a six-qubit variational quantum circuit (VQC) with a CNN architecture, we hope to leverage the strengths of both worlds: the feature extraction strength of deep learning and the non-classical processing strength of quantum computing. This research work presents a solution for defect detection that hopes to find a balance between accuracy and efficiency, hoping to fill the gaps in data efficiency and feature representation. We will present new experimental results on the performance of HQCNN models for industrial quality assurance, and will try to bridge the scientific and practical gaps in current developments by reviewing the current advances in the field. By combining innovations in AI and quantum machine learning and applying them to the requirements of fabric and steel production, we hope to contribute to the advancement of intelligent, reliable, and economically feasible quality assurance systems for future industrial settings.

### 1.1 Problem statement

In contemporary manufacturing, quality standards are stricter than ever and traditional manual inspection methods remain slow, error-prone, and unsuitable for handling complex materials such as fabrics and metals. With increasing pressure to meet strict inspection deadlines, engineers are turning to faster and more accurate automated systems. Deep learning has already improved defect detection; however, conventional networks often struggle with complex fault patterns and demand large, well-annotated datasets to achieve high accuracy. To bridge these challenges, our research comes up with a hybrid approach that combines quantum computing with traditional deep learning. The quantum-classical model aims to improve both accuracy and robustness, offering a more effective solution for automated defect detection in materials.

### 1.2 Research gaps

Despite using advanced methods in automated defect detection, some drawbacks still prevail. Comprehensive and representative datasets are often considered as important, yet there is limited guidance on how to effectively collect or expand image datasets to capture the diversity of material types and defects found in industrial settings. Building and augmenting data that includes both common and rare defect patterns remains a constant challenge. While hybrid approaches involving classical methods and automated systems are being proposed nowadays, a rigorous comparison across different products, classes and defects is still rare. The economic and operational feasibility of automated inspection is often neglected, particularly in relation to cost structures, resource requirements, ease of integration into existing production lines, scalability, and return on investment. Without proper consideration of these factors, the dispersal of such technologies across the sector remains restricted.

Furthermore, existing Quantum Machine Learning (QML) frameworks primarily rely on static-depth quantum circuits implemented using standard libraries. This static approach creates a computational bottleneck in the NISQ era: applying maximum circuit depth to every image wastes coherence time, while shallow circuits fail on complex features. Moreover, early studies rarely provide rigorous ablation studies comparing quantum networks against structurally equivalent classical bottlenecks, leaving the true source of their performance gains ambiguous. This work fills this gap by introducing the Gumbel-Softmax dynamic depth controller as a fundamental structural innovation. This data-adaptive quantum architecture is rigorously benchmarked against parameter-matched classical alternatives to prove genuine quantum feature advantages.

### 1.3 Contributions

This paper adds up a contribution to the development of automated defect detection in materials, especially targeting fabric and steel surfaces. From existing literature review, the advancement of inspection methods from labor-intensive manual approaches to modern AI-enabled techniques is traced by this study. A particular emphasis is put on developments in machine learning and computer vision, especially CNNs and their combinations to improve detection accuracy. A key component of this work is the proposed HQCNN architecture which is designed to integrate quantum computing with deep learning to capture fine-grained defects. By using the fabric and metal surface defect datasets, performance of HQCNN against classical ResNet-18 benchmarks and fixed-depth quantum baselines is evaluated. To assess the efficiency gains of the dynamic depth mechanism, ablation studies are conducted. This work discusses the key challenges in the field, including the need for large and diverse datasets, refinement of hybrid approaches, and economic feasibility of deploying advanced AI models in manufacturing environments. The paper provides pragmatic insights for researchers and industry by identifying these barriers and suggesting future directions, supporting the development of reliable and efficient quantum enhanced defect detection mechanism.

### 1.4 Structure of the paper

Rest of the paper is structured as follows. Section 2 contains the literature review, which examines recent advancements in quantum convolutional neural networks, hybrid architectures, and their use in automated inspection. Section 3 comprises of the proposed methodology, which describes the Hybrid Quantum-Classical Neural Network (HQCNN) architecture, implementation of the dynamic depth controller, and design of the variational quantum circuit. Section 4 describes the experimental framework, covering dataset characteristics, evaluation metrics, and result analysis on fabric and steel defects. Section 5 summarizes the key findings and outlines potential directions for future research.

## 2 Literature review

Quantum convolutional neural networks (QCNNs) use quantum concepts in identifying crops diseases and perform better than classics CNNs in diagnosing the corn leaf disease. They can more easily obtain complex features by applying quantum superposition and entanglement, with a maximum accuracy of 99.0 Percent, increased precision and reduced loss. This renders QCNNs as a prospective solution to early and accurate detection of diseases in agriculture (Arepalli et al., 2025). Quantum machine learning models effectively classify flowers by analyzing color, texture, and structural features. They demonstrated strong potential for scalable agricultural quality assessment by employing quantum-inspired algorithms to achieve 100.0% accuracy with increased efficiency (Cauvery et al., 2025). By integrating quantum concepts like superposition and entanglement into neural networks, hybrid CNN-QCNN models improve multiclass image classification. After testing on Modified National Institute of Standards and Technology (MNIST) and CIFAR-10, they achieved very high accuracies (99.8 and 98.3%) with loss lower than classical CNNs which showed a strong potential for future image classification tasks (Golchha et al., 2023). HQCNNs combine quantum convolutional layers with classical networks for facial emotion recognition. Using data re-uploading and ensemble strategies, they enhance feature extraction and classification reliability. Despite hardware and data limitations, they achieve a competitive 70.25% accuracy, showing the practical potential of quantum-classical models (Khurelsukh, 2022). Hybrid quantum-classical models are gaining attention in medical imaging. A Canadian Institute for Advanced Research (QCCNN) integrating VQC layers with ResNet-18 for dementia detection from MRI scans encodes features into quantum space for better representation. Tested on a large Kaggle dataset, it achieved 98.9% accuracy, outperforming classical ResNet-18 (94.4%), with improved precision, recall, and F1-score. Statistical tests confirmed the gains, showing strong potential for quantum-enhanced medical diagnosis (Kim, 2023). Scalability issues in the noisy intermediate-scale quantum (NISQ) era are tackled using dynamic quantum circuits with mid-circuit measurements and classical feedback. Two strategies are used: iterative compilation, which reuses few qubits but increases runtime, and parallel distributed compilation, which reduces runtime but adds gate overhead. The optimal choice depends on the algorithm and hardware noise, as no single approach fits all cases (Kole et al., 2024). Quantum machine learning has made significant progress across domains like cybersecurity, finance, healthcare, and drug discovery. The most realistic strategy in the NISQ era is to use hybrid quantum-classical models, with techniques like Quantum Support Vector Machine (QSVMs) and quantum neural networks (QNNs) demonstrating practical potential. To fully realize QML's potential, however, issues like noise, qubit limitations, and high costs must be resolved through advancements in hardware, error correction, and algorithms (Lamichhane et al., 2025). For time-series classification, HQResNet is a hybrid quantum-classical model that incorporates residual blocks based on QCNN into a ResNet architecture. Without re-uploading data, classical layers control data dimensions and get around no-cloning restrictions. Compared to classical and other quantum models, experiments on Ultra-Wideband (UWB) Line-of-Sight/Non-Line-of-Sight (LOS/NLOS) classification demonstrate higher accuracy with a significantly smaller number of parameters. The design effectively makes use of entanglement and quantum parallelism while maintaining noise resilience (Noh et al., 2025). Prior to classical classification, the method efficiently extracts features from compressed images by encoding them into quantum gates using a dynamic trap module. Although there are still issues with limited qubits, decoherence, and algorithm design, quantum and photonic computing hold great promise for image processing (Nouioua and Yahiaoui, 2023). Quantum computing can transform image processing by enabling faster computation and efficient data representation through superposition and entanglement. Photonic quantum computing is especially promising due to reduced decoherence, and early applications like quantum edge detection show practical potential. However, limitations in qubit availability, stability, and specialized algorithms must be addressed to enable large-scale adoption (Oh et al., 2020). A review of 94 studies from 2017 to 2023 shows major progress in quantum machine learning across algorithms like QNNs, QSVMs, and QGANs, with applications in vision, NLP, finance, healthcare, and chemistry. Despite current hardware limits, hybrid quantum-classical models are effective, and continued advances suggest practical quantum advantage is increasingly feasible (Peral-García et al., 2024). Quantum convolutional neural networks enhance computer vision by using quantum parallelism and entanglement to improve feature extraction and efficiency. Classical CNN layers are mapped to parameterized quantum circuits, enabling familiar architectures in a quantum setting. Although challenges remain in hardware access, nonlinearity, and data encoding, QCNNs show strong potential across vision, signal processing, pharmaceuticals, and cryptography as quantum technology matures (Rajesh et al., 2021). Reejisha and Mohan (2023) presented a hybrid quantum-classical image classification framework using QNNs and QCNNs. Fashion-MNIST images were reduced to 4 × 4, encoded into qubits, processed through parameterized quantum circuits, and classified using classical layers. Implemented with TensorFlow Quantum and Cirq, the work demonstrates a practical end-to-end hybrid modeling approach (Reejisha and Mohan, 2023). Quantum machine learning using QNNs and QCNNs has shown potential for image classification on the Fashion-MNIST dataset. With images reduced to 4 × 4 and implemented using TensorFlow Quantum and Cirq, QNNs achieved 90.0% accuracy and QCNNs 86.0%. Although classical CNNs still perform better, these results demonstrate an important step toward effective quantum-classical integration as hardware and algorithms improve (Reejisha and Mohan, 2023). ResNet50 is a deep image classification model with residual connections to prevent vanishing gradients. It processes 224 × 224 images through convolutional and bottleneck layers, producing class probabilities via fully connected layers. It achieves high accuracy and is widely used, with performance enhanced through transfer learning (Singla et al., 2024). Quantum circuit learning variational quantum algorithms have been used to solve dynamical equation on noisy quantum computing devices. The parameter shift rule is used by parameterized circuits to compute derivatives, and are scaled poorly to larger systems because of noise. Basic computations were solved successfully, both indicating future and real-life limitations in the NISQ era (Schillo et al., 2025). Quantum Classical Convolutional Neural Network (QuCNN) specifically uses Swap gate test (SWAP) tests to extract features and backpropagation with entanglement-based gradient calculation using ancilla qubits to adapt classical CNNs to quantum computing. It is tested on the MNIST, and it proves that parameterized quantum filters can be learned with the help of shallow quantum circuits effectively (Stein et al., 2022). Comparative studies of classical CNNs and quantum CNNs (QCNNs) for binary image classification showed that QCNNs outperformed classical models, specifically when there is class imbalance. On evaluation of same datasets, QCNNs achieved up to 94.4% accuracy and 0.94 F1-score, compared to 80.0% accuracy and 0.79 F1-score for CNNs. The results were improved scalability and performance of quantum convolutional layers that indicated strong potential for advancing deep learning as quantum hardware improved (Tasnim et al., 2025). Classical concepts are extended by quantum circuits using qubits, which utilize superposition and entanglement to depict multiple states at the same time. Quantum gates like Hadamard and CNOT build circuits and protocols such as quantum teleportation enable advanced algorithms, which lays the foundation for modern quantum applications (Vasantrao et al., 2025). A research by Zhang et al. (2024) presents an innovative methodology for developing adaptive quantum layer architectures, a hybrid neural network was introduced featuring dynamically customized quantum circuit architectures customized to specific input images. The main component of their model employs a Parameterized Quantum Circuit (PQC) that functionally replaces traditional convolutional layers, enabling input-specific quantum processing adaptations (Zhang et al., 2024).

MG-QCNN is a quantum machine learning model for face recognition that involves variational circuits with multigate entanglement and rotation gates for effective feature extraction. It achieves up to 96.0% accuracy when tested on Yale and ORL datasets, outperforming classical and earlier quantum models while using few qubits, emphasizing its potential for biometrics and medical imaging (Zhu et al., 2024). Variational quantum circuits are applied to malaria diagnosis from blood cell images with the help of a quantum-classical hybrid approach. VQCs classified optimized RBC features with fewer parameters than classical methods, and combined with a rule-based system, achieved over 99.0% accuracy, showing strong potential for quantum-enhanced medical imaging (Hossain et al., 2021). Stable fluorescent sulfur quantum dots (CMC-SQDs) synthesized from carboxymethyl cellulose offer high stability and low cytotoxicity for live cell imaging. They also serve as sensitive fluorescent probes for detecting Cr(VI) and ascorbic acid, highlighting their versatility in bio-imaging and biochemical sensing (Duan et al., 2020). Using hybrid quantum-classical lookup tables and optimized circuits, a quantum fuzzy inference engine has been modified for edge detection on NISQ hardware. With fewer operations, it achieves performance comparable to classical techniques like Canny and Sobel, proving the viability of quantum fuzzy logic for image processing (Nunziata et al., 2025). A quantum splitting CNN is used in the distributed quantum disease detection (DQDD) model to split large circuits among several quantum devices. When tested on skin cancer datasets, it addresses hardware limitations in complex disease detection by capturing both high- and low-dimensional features while lowering qubit and entanglement requirements (Li et al., 2025). High-resolution visualization of biological structures and early biomarker detection are made possible by AI-enhanced quantum imaging, which improves nanosensing technologies like plasmonic biosensors and quantum dots. This integration supports preventive healthcare by providing more precise, non-invasive medical diagnostics (Gurjar et al., 2025). To categorize retinopathy of prematurity stages, QMVIT uses MobileNet blocks, vision transformers, and quantum principles. It is appropriate for low-resource neonatal healthcare devices due to its lightweight hybrid design, which achieves high accuracy while preserving a minimal quantum trace (Sankari et al., 2025). According to recent surveys, quantum image processing outperforms classical methods by utilizing entanglement and superposition. Faster image representation and processing are provided by QIP algorithms, such as quantum edge detection and optimisation, which have the potential to achieve exponential speedups for computationally demanding tasks (Ranjan et al., 2020). A quantum-inspired adaptive loss detection method using reinforcement learning and a network-in-network architecture restores images in optical quantum environments. It effectively clarifies and preserves image accuracy in real-time, outperforming traditional metrics, and advancing applications in surveillance and smart cities (Priyanka et al., 2024). Security in quantum image processing has also seen new developments with the proposal of an encryption algorithm based on a 3D chaotic system and the quantum Rubik's cube principle. This method employs chaotic key sequences to control quantum permutation and diffusion operations within the Novel Enhanced Quantum Representation (NEQR) representation model. The resulting encryption scheme effectively resists statistical attacks and noise interference while significantly reducing time complexity compared to classical counterparts (Song et al., 2024). A variational quantum algorithm (VQA)-based image classification framework replaces classical global pooling with a variational quantum circuit to retain fine-grained spatial features. Experiments show it reduces parameters while improving accuracy and F1 scores by capturing more discriminative details (Chen, 2024). A hybrid model combining HQCNN, BiLSTM, and MobileNetV2 has been developed for skin cancer detection. It merges quantum feature extraction with temporal context capture, achieving robust classification of malignant and benign lesions while enhancing generalization and computational efficiency for clinical use (Hussein et al., 2025). By 2026, additional examples of hybrid quantum-classical frameworks had emerged across diverse application domains. In the same year, Verdone introduced Hybrid Quantum Neural Networks (HQNNs) that combine autoencoders (AEs) with quantum neural networks (QNNs) for cardiovascular disease diagnosis, as presented in their work published in Verdone et al. (2026). Their framework employs classical autoencoders to compress high-dimensional medical data into a compact latent representation, which is subsequently processed by a quantum neural network. This separation between classical and quantum components enables the quantum model to operate on information-rich yet reduced-dimensional encodings, thereby lowering quantum resource requirements and making the approach suitable for noisy intermediate-scale quantum (NISQ) devices. The authors reported a classification accuracy of approximately 90.98% on the Cleveland heart disease dataset, outperforming several existing classical and quantum-based approaches. Furthermore, experimental evaluations conducted on IBM quantum hardware demonstrated that the proposed HQNN framework remained robust and reliable even when trained with limited datasets. Despite these advantages, the study also highlighted certain architectural limitations associated with the quantum circuit design. In particular, the framework utilized a fixed number of ansatz layers *l* as a global hyperparameter, implying that every sample within the dataset was processed with the same quantum computational complexity. Consequently, the architecture lacked adaptive mechanisms for dynamically increasing or decreasing circuit depth according to the complexity of individual samples. In the domain of structural engineering, Sukulthanasorn and Terada (2026) proposed a novel topology optimization framework based on quantum neural networks utilizing parameterized quantum circuits (PQCs). Their work, published in Sukulthanasorn and Terada (2026), demonstrated the growing applicability of hybrid quantum-classical learning architectures beyond traditional classification problems and into computational engineering optimization tasks. For the first time, their framework demonstrated PQC model-based pattern prediction and learning, while also showing that quantum neural networks can be integrated into iterative topology optimization workflows extending from coarse training toward a wide range of two-dimensional and three-dimensional structural problems, including variations in boundary conditions, high-resolution meshes, and volumetric constraints. When compared with conventional gradient-based optimization approaches, the quantum-assisted framework achieved nearly a 90% reduction in computational time. This result highlights the potential quantum advantage in engineering design optimization tasks. However, the PQC employed within the framework utilized a fixed-depth circuit architecture. Consequently, every iteration in the optimization process retained the same number of variational layers irrespective of the structural complexity of the input sample. As a result, the framework applied an identical quantum processing budget to all topological configurations, regardless of whether the structures were simple or highly complex. This could lead to unnecessary gate operations for simpler topologies, while simultaneously limiting the representational capability of the circuit for more intricate structural samples. Furthermore, the model lacked an adaptive depth adjustment mechanism capable of dynamically modifying quantum resource utilization according to varying levels of structural complexity. In the geospatial domain, Mahendra et al. (2026) proposed a hybrid network incorporating quantum convolutional neural networks (QCNNs) for the classification of remote sensing images using multispectral LISS-III satellite imagery. By integrating principles of quantum computation with classical deep learning techniques, the proposed QCHNet framework addressed the longstanding challenge of handling complex spectral and spatial variations present in Earth observation datasets. A comparative study on land-use and land-cover (LULC) classification demonstrated that QCHNet achieved an overall classification accuracy of 96.54%. The performance of QCHNet significantly exceeded that of a classical CNN baseline, which achieved an accuracy of 90.70%, as well as a standard QCNN model with an accuracy of 94.27%. In addition, QCHNet reported a precision of 96.4% and a recall of 97.1%, along with a comparable F1-score. These results reinforce the capability of quantum-enhanced hybrid architectures to improve the classification of spectrally overlapping land-cover classes, which often present substantial challenges for purely classical approaches. However, the quantum convolutional layers within QCHNet employed a rigid circuit structure in which the same circuit depth was uniformly applied to all input patches regardless of their spectral complexity. In practical satellite imagery, different regions may exhibit substantially different levels of complexity. For instance, homogeneous water bodies are comparatively easier to classify, whereas heterogeneous urban regions mixed with agricultural boundaries may require significantly richer feature representations. The absence of patch-level adaptivity therefore results in inefficient quantum resource utilization, as simpler patches consume the same quantum computational budget as highly complex regions that require deeper representational capacity. In spite of notable progress in quantum convolutional networks and hybrid quantum-classical architectures across medical imaging, agriculture, and image classification there are still unresolved challenges regarding real-world industrial defect detection. Comparative analysis on land-use and land-cover (LULC) classification demonstrated that QCHNet achieved an overall classification accuracy of 96.54%. In comparison with other approaches, QCHNet outperformed a classical CNN baseline, which achieved an accuracy of 90.70%, as well as a standard QCNN model with an accuracy of 94.27%. Furthermore, the framework reported a precision of 96.4%, a recall of 97.1%, and a comparable F1-score. These findings illustrate the capability of quantum-enhanced hybrid architectures to improve the discrimination of spectrally overlapping land-cover classes, a task that often remains challenging for purely classical models.

Despite these advantages, the quantum convolutional layers within QCHNet utilized a fixed circuit architecture in which identical circuit depths were applied uniformly across all input patches irrespective of their spectral complexity. In practical remote sensing scenarios, satellite image patches may contain regions with highly imbalanced informational complexity. For instance, homogeneous regions such as large water bodies are comparatively easier to classify, whereas heterogeneous urban–agricultural regions require significantly more expressive feature representations. Since all image patches were processed using the same quantum circuit configuration, the framework exhibited notable structural inefficiency. The absence of an adaptive depth mechanism implies that relatively simple and easily classifiable patches consumed the same quantum computational resources as highly complex patches requiring deeper representational capacity. Most existing approaches rely on fixed-depth or statically designed quantum circuits, often underutilizing quantum expressivity for complex samples or imposing unnecessary computational overhead for simpler inputs. Prior studies frequently evaluate performance on reduced resolution or synthetic datasets, offering limited evidence of scalability to high variability. Additionally, the majority of hybrid models prioritize accuracy gains, with comparatively giving little attention to quantum resource efficiency, adaptive circuit design, or deployment feasibility in practical manufacturing pipelines. To overcome such limitations, the present work advances the field by introducing a resource aware Hybrid Quantum Classical Neural Network (HQCNN) incorporating data adaptive quantum circuit depth selection. The proposed framework balances representational power and quantum efficiency by integrating a fine-tuned ResNet-18 backbone for classical feature extraction with a dynamically controlled variational quantum circuit and a depth penalized training objective. Such design addresses the rigidity and scalability constraints, and practical gaps identified in prior research, establishing balanced progression toward a more adaptable, industrially viable quantum enhanced defect detection technique.

## 3 Methodology

### 3.1 Proposed HQCNN architecture

**Figure 1:**
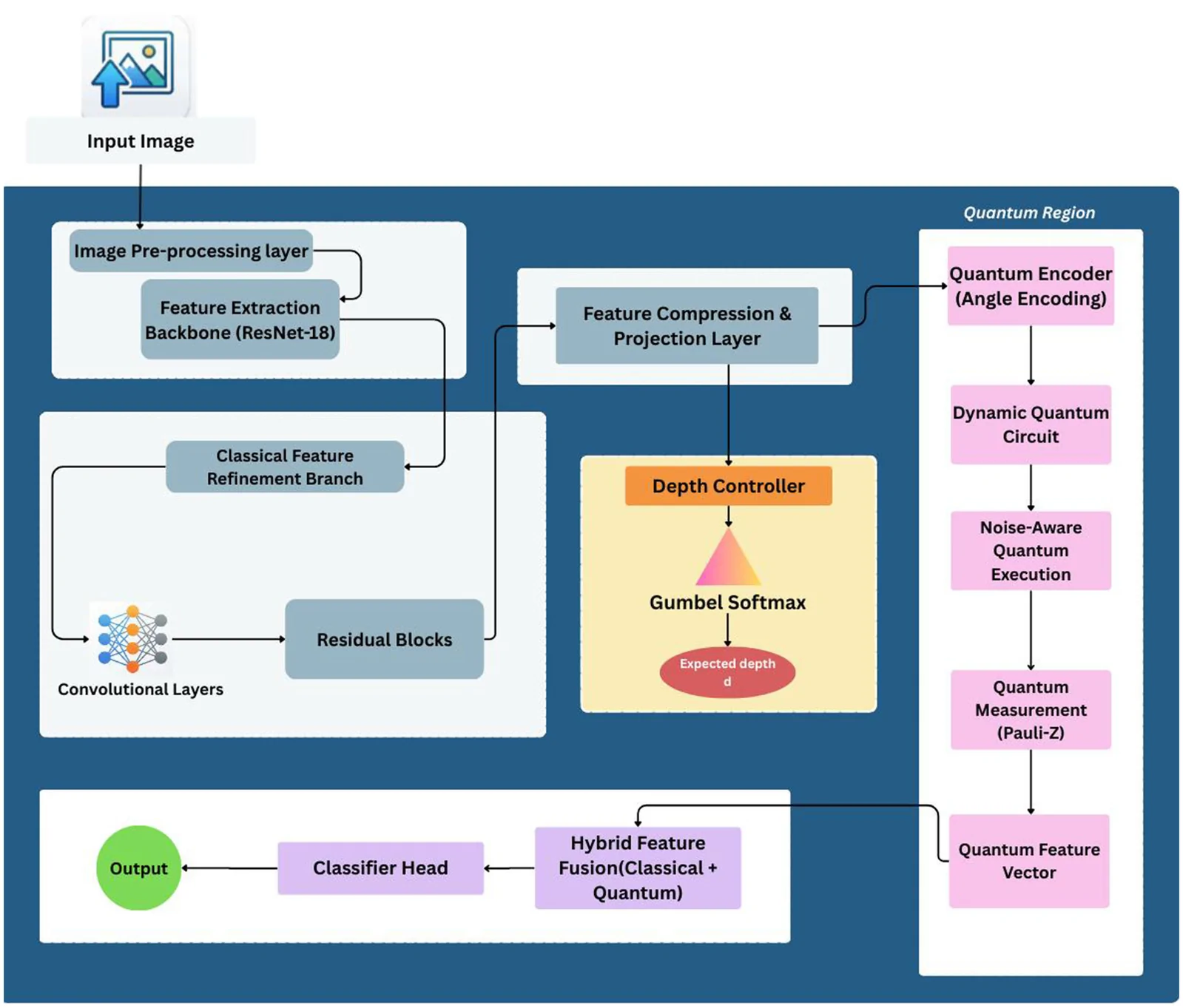
Defect detection architecture. The flowchart illustrates the complete integration of the ResNet-18 classical feature extraction backbone, the Gumbel-Softmax-driven dynamic depth controller, and the noise-aware variational quantum circuit for hybrid defect classification.

The Hybrid Quantum Classical Neural Network (HQCNN) illustrated in Figure 1, is a multiphase structure that is targeted at the effective classification of defects. The pipeline begins by a backbone that is a ResNet-18, which has been pre-trained on ImageNet before undergoing fine-tuning, which can be considered as an effective feature extractor. This serves as a backbone and the input images are converted to high-dimensional classical feature vectors which encode the complex, hierarchical visual patterns that are of significance to the defect detection problem. The classical feature-vector is then further toward the heart of the architecture: a dynamic quantum processing unit. This unit is made up of two major parts that work as a unit. The features are then analyzed by a compact neural sub-network, which is a learnable depth controller, to identify an optimal circuit depth on the input and can be trained using the Gumbel-Softmax reparameterization trick to provide end-to-end differentiability. At the same time, the classical features are encoded in a six-qubit variational quantum circuit (VQC) with the help of angle embedding. The VQC consists of repeating layers of RY/RZ rotation gates, and a ring of CNOT entangling gates, but its most important property is its data-adaptive depth, with the number of layers independently determined by the controller. After quantum evolution, a quantum feature vector is measured out of *Z*-basis expectation values. These quantum characteristics are then added to the original classical feature vector through concatenation, to form a hybrid representation that is even rich in learned classical patterns and may have quantum dependencies. The resulting fused vector is then fed into a last high density classifier to produce the final prediction. A resource-aware objective function is used to train the entire architecture. This operation is the best predictor while utilizing a penalty term that is proportional to the predicted quantum circuit depth, which is the sole purpose of the models to act in the interest of high predictive precision and efficiency in quantum resource consumption, which will be central to near-term hardware.

### 3.2 Dynamic variational quantum circuit (VQC)

**Figure 2:**
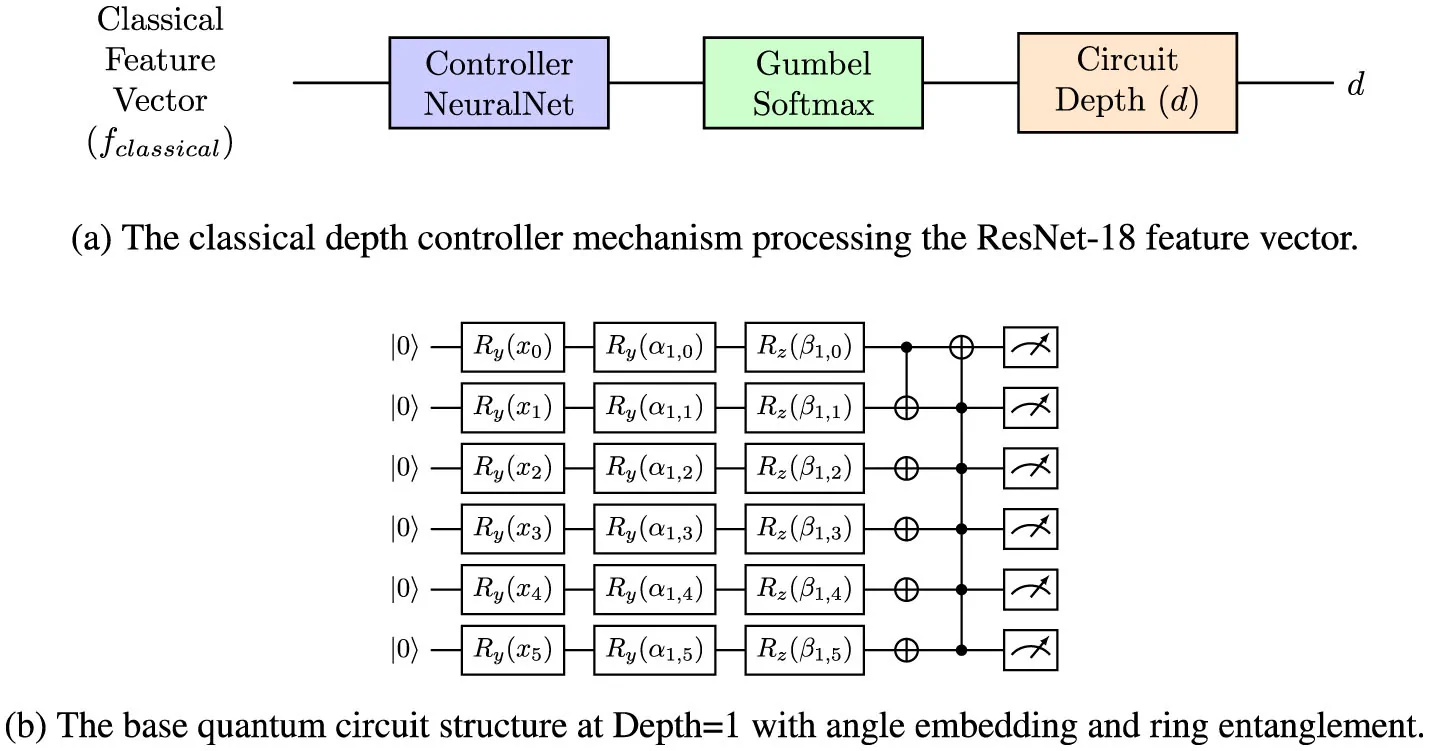
The hybrid architecture components. **(a)** Details the controller network utilizing Gumbel-Softmax to predict discrete depths. **(b)** Illustrates the foundational six-qubit VQC configuration mapping classical features to quantum states.

**Figure 3:**
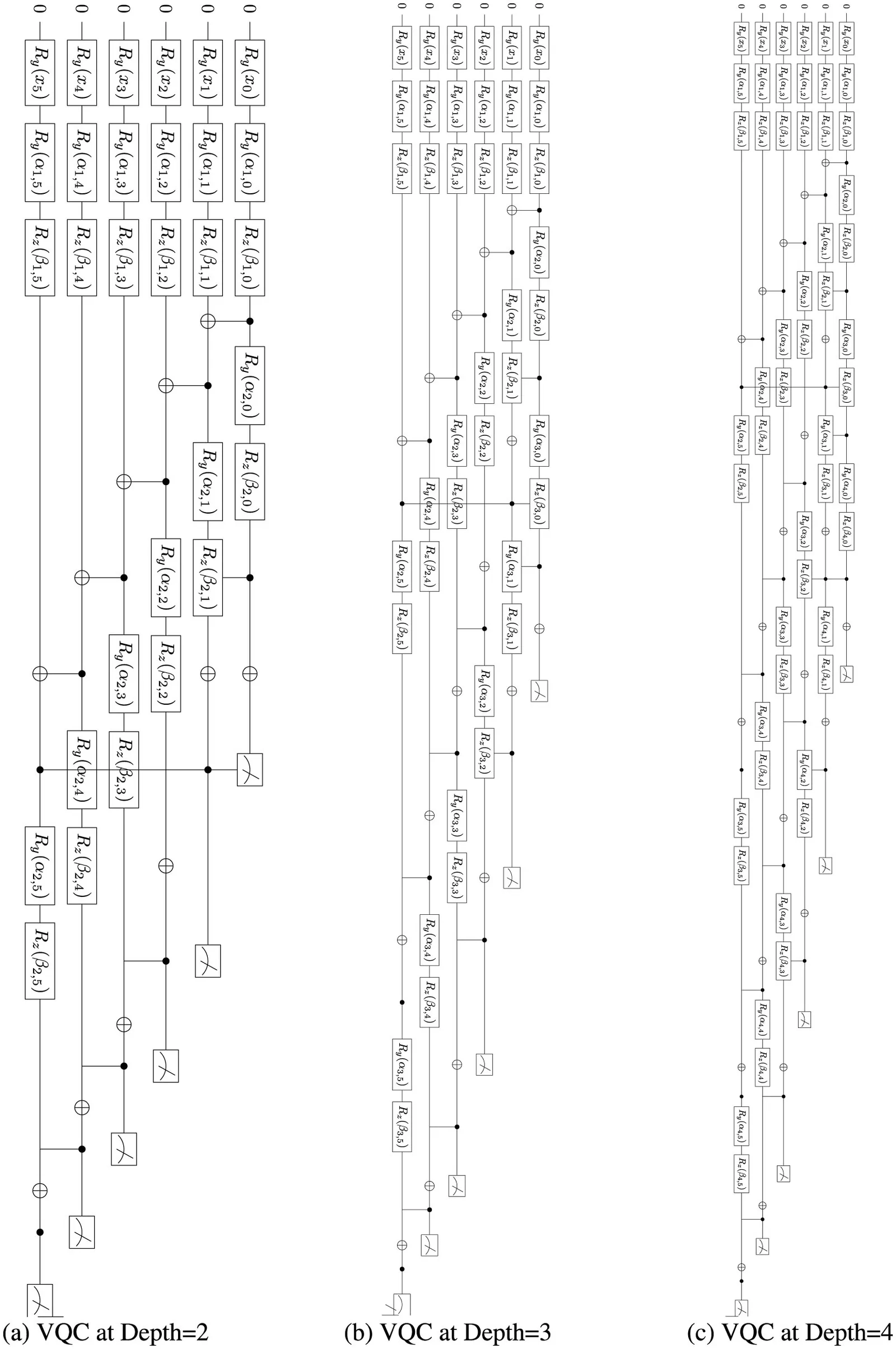
Evolution of VQC Architectures demonstrating the scalability of the quantum layer. **(a)** Depth = 2 with two stacked parameterized layers. **(b)** Depth = 3 showcasing increased parameter counts for complex feature mapping. **(c)** Depth = 4 with expanded layers representing the maximum expressivity allowable by the dynamic controller.

Dynamic depth capability of the VQC is controlled by a classical depth controller, the workflow of which is shown in Figure 2a. It is a tiny neural sub-network which decides the complexity of the circuit required on each sample of the input. As its input, it takes the high-dimensional classical feature vector of the ResNet backbone (*f*_*classical*_). These features are fed into the Controller NeuralNet which generates a set of logits, which is then run through a Gumbel-Softmax layer. This operation gives a differentiable method to sample an integer value of a discrete value, which will be the depth of that particular sample of the chosen circuit (d). The mechanism of this model enables it to learn an optimal data-adaptive policy of quantum resource allocation versus representational power. The fundamental quantum unit of the HQCNN is a six-qubit dynamic variational quantum circuit (VQC), the architecture of which is made to be expressive and flexible. A single layer of depth D = 1 is the basic unit of the circuit as in Figure 2b. This base layer achieves three key functions: encoding of data with the help of gates of the form of *R*_*y*_(*x*_*i*_) more precisely, a trainable variational transformation with the help of the gates of type *R*_*y*_(α) and *R*_*z*_(β) and entanglementing between multiple qubits in a ring of CNOT gates. To scale the complexity of circuit, this layer is stacked vertically as in the addition of the D = 2 configuration to Figure 3a. To demonstrate the increase in the representational power in detail, more complicated circuits of Depth = 3 and Depth = 4 are shown in Figures 3b, c, respectively. With increased depth, the size of the trainable parameters and entanglement operations increase exponentially enabling the VQC to learn more complex correlations between data. The HQCNN is based on this versatile design, which allows the classical depth controller to choose the most suitable complexity of the circuit on a per-sample scale and thus achieve a balance between the performance and quantum resource efficiency.

### 3.3 Model implementation and training

Algorithm 1 illustrates the HQCNN structure workflow that connects classical deep learning with adaptive quantum processing. The process begins with classical feature extraction (Step 1) is carried out by a fine-tuned ResNet-18 which extracts hierarchical visual patterns from the images, as defined in Equation 1. We then apply feature compression (Step 2) via Equations 2, 3 to map these high-dimensional features onto the limited qubits of the NISQ hardware. The choice of exactly six qubits was made to optimally balance the representational capacity required for the compressed feature space with the simulation constraints and coherence limitations typical of noisy intermediate-scale quantum (NISQ) devices. Scaling to larger datasets or more complex features could theoretically involve increasing the qubit count; however, this currently incurs exponential computational overhead on classical state-vector simulators and heightened susceptibility to decoherence on physical QPUs. A key innovation is the Adaptive Depth Selection mechanism (Step 3, Equation 4). Here, a lightweight controller evaluates the input complexity to sample a discrete integer depth *d* for execution, while simultaneously computing the expected depth to enforce resource efficiency during training. This discrete depth *d* configures the quantum circuit execution (Step 4), where features are encoded and processed through variational unitaries (Equations 5, 6). Finally, the quantum measurements are fused with the classical features (Step 5) to build a unified hybrid embedding for classification (Step 6, Equation 8).

Algorithm 1 Hybrid quantum classical neural network (HQCNN). 
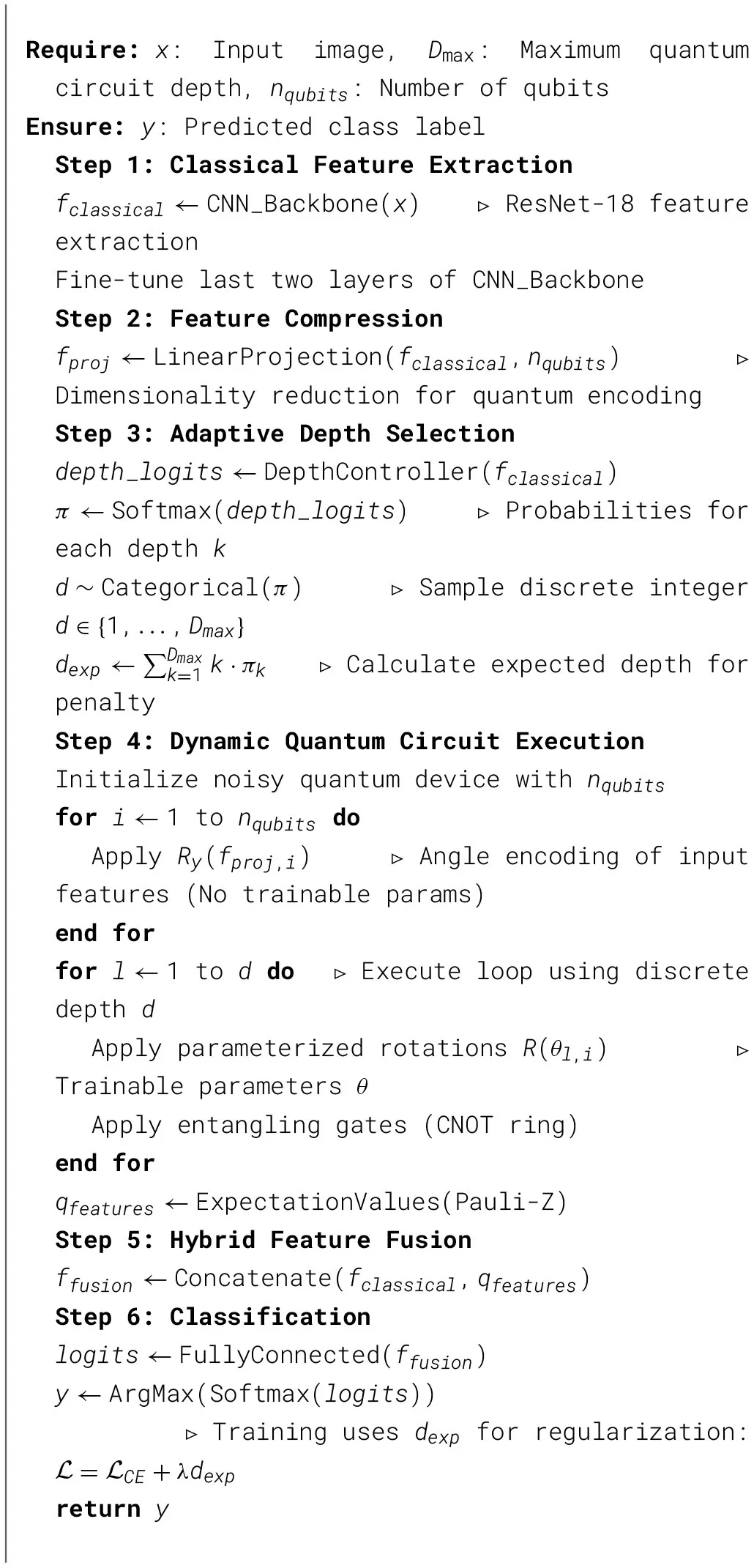

Equation 1 shows that a feature vector f is created by passing the input data denoted as x, through a ResNet18 convolutional neural network (CNN) after that we get a resulting vector f of 512-dimensions.


$$\begin{array}{l} f=\text{ResNet18}\left(x\right),\;f\in {\mathbb{R}}^{512} \end{array}$$ (1)

Equation 2 takes input of extracted feature vector *f* from the previous step is mapped into a new vector *f*′ with a smaller dimension, *n* (where *n* equals the number of qubits used in HQCNN). This is implemented by a simple linear transformation using a weight matrix *W*_prep_ and a bias vector *b*_prep_. This process is a prerequisite for subsequent steps.


$$\begin{array}{l} f^{\prime }=W_{\text{prep}}f+b_{\text{prep}},\;f^{\prime }\in {\mathbb{R}}^{n} \end{array}$$ (2)

Equation 3 depicts a non-linear transformation to prepare the feature vector for quantum encoding. Values are compressed into the range [−1, 1] by the tanh function, and is scaled by $\frac{\pi }{2}$ to ensure that the transformed features fit within valid rotation angles for quantum gates.


$$\begin{array}{l} \tilde{f}=\frac{\pi }{2}\tanh\left(f^{\prime }\right) \end{array}$$ (3)

Equation 4 formulates the dynamic depth controller. A linear layer transform the classical features *f* to a probability distribution π over possible depths, and the discrete depth *d* is sampled using the Gumbel-Softmax relaxation to ensure differentiability throught training process.


$$\begin{array}{l} \pi =\text{Softmax}\left(W_{c}f+b_{c}\right),\;d\sim \text{Gumbel}\left(\pi \right) \end{array}$$ (4)

Equation 5 shows the encoding of classical data into a quantum state using angle embedding with the *R*_*y*_ rotation gate.


$$\begin{array}{l} R_{y}(\tilde{f})|0\rangle =\cos(\frac{\tilde{f}}{2})|0\rangle +\sin(\frac{\tilde{f}}{2})|1\rangle \end{array}$$ (5)

Equation 6 describes the variational quantum circuit consisting of parameterized rotation gates (*R*_*y*_) and entangling CNOT gates arranged in a ring topology. The number of layer repetitions is determined by the dynamic depth *d*, and the parameters θ are optimized during training.


$$\begin{array}{l} U\left(\theta \right)=\overset{d}{\underset{l=1}{\prod }}\left(\underset{i}{\prod }R_{y}\left(\theta_{l,i}\right)\cdot \underset{i}{\prod }\text{CNOT}\left(i,i+1\right)\right) \end{array}$$ (6)

Equation 7 determines the measurement of each qubit in the computational basis using the Pauli-Z operator. The expectation values *z*_*i*_ serve as classical features derived from quantum states for downstream classification.


$$\begin{array}{l} z_{i}=\langle \phi \left(\theta ,\tilde{f}\right)|Z_{i}|\phi \left(\theta ,\tilde{f}\right)\rangle \end{array}$$ (7)

Equation 8 represents the hybrid classification stage. The quantum measurement outcomes *z* are concatenated with the original classical features *f* and transformed through a linear layer with ReLU activation to generate hidden features *h*, followed by a softmax layer that produces the predicted class probabilities ŷ.


$$\begin{array}{l} h=\text{ReLU}\left(W_{q}\left[z⊕f\right]+b_{q}\right),\;ŷ=\text{Softmax}\left(W_{o}h+b_{o}\right) \end{array}$$ (8)

Equation 9 is used to create a balance between high classification accuracy and quantum resource efficiency. The total loss, L, combines the standard categorical cross-entropy loss ($L_{CE}$) with a regularization term that penalizes the expected circuit depth (*𝔼*[*d*]). A hyperparameter λ≥0 controls the trade-off between these two competing goals.


$$\begin{array}{r} \begin{array}{c} \;L=L_{CE}+\lambda 𝔼\left[d\right]\\ \text{where, }L_{CE}=-\overset{N}{\underset{i=1}{\sum }}y_{i}\log\left({ŷ}_{i}\right)\\ \text{and, }𝔼\left[d\right]=\overset{D_{max}}{\underset{k=1}{\sum }}k\cdot \pi_{k} \end{array} \end{array}$$ (9)

Equation 10 expresses the gradient of the loss function with respect to the quantum circuit parameters θ. It is computed using the chain rule of derivatives in the backpropagation algorithm.


$$\begin{array}{l} \nabla_{\theta }L=\frac{\partial L}{\partial ŷ}\cdot \frac{\partial ŷ}{\partial h}\cdot \frac{\partial h}{\partial z}\cdot \frac{\partial z}{\partial \theta } \end{array}$$ (10)

Equation 11 shows the parameter-shift rule used to compute gradients in variational quantum circuits. This method evaluates the expectation value of an observable *O* at shifted parameter values ($\theta \pm \frac{\pi }{2}$) to estimate the derivative efficiently.


$$\begin{array}{l} \frac{\partial \langle O\rangle }{\partial \theta }=\frac{1}{2}\left({\langle O\rangle }_{\theta +\frac{\pi }{2}}-{\langle O\rangle }_{\theta -\frac{\pi }{2}}\right) \end{array}$$ (11)

## 4 Performance evaluation

A thorough experimental evaluation was carried out to validate the effectiveness and strength of the proposed Hybrid Quantum Classical Neural Network (HQCNN).The study examines how well the model detects and classifies the defects across two different industrial domains of textile manufacturing and metal surface inspection. This section begins by outlining the experimental framework and describing the characteristics of the benchmark datasets specifically the fabric defect dataset and the metal surface defect dataset. Subsequently, the quantitative metrics used to measure classification performance are accuracy, precision, recall, F1-score, ROC-AUC, MCC and Cohen's Kappa are defined. Finally, a detailed analysis of the results is presented to demonstrate the superior defect detection capabilities of the dynamic quantum approach compared to traditional classical and static quantum baselines.

### 4.1 Experimental setup

**Figure 4:**
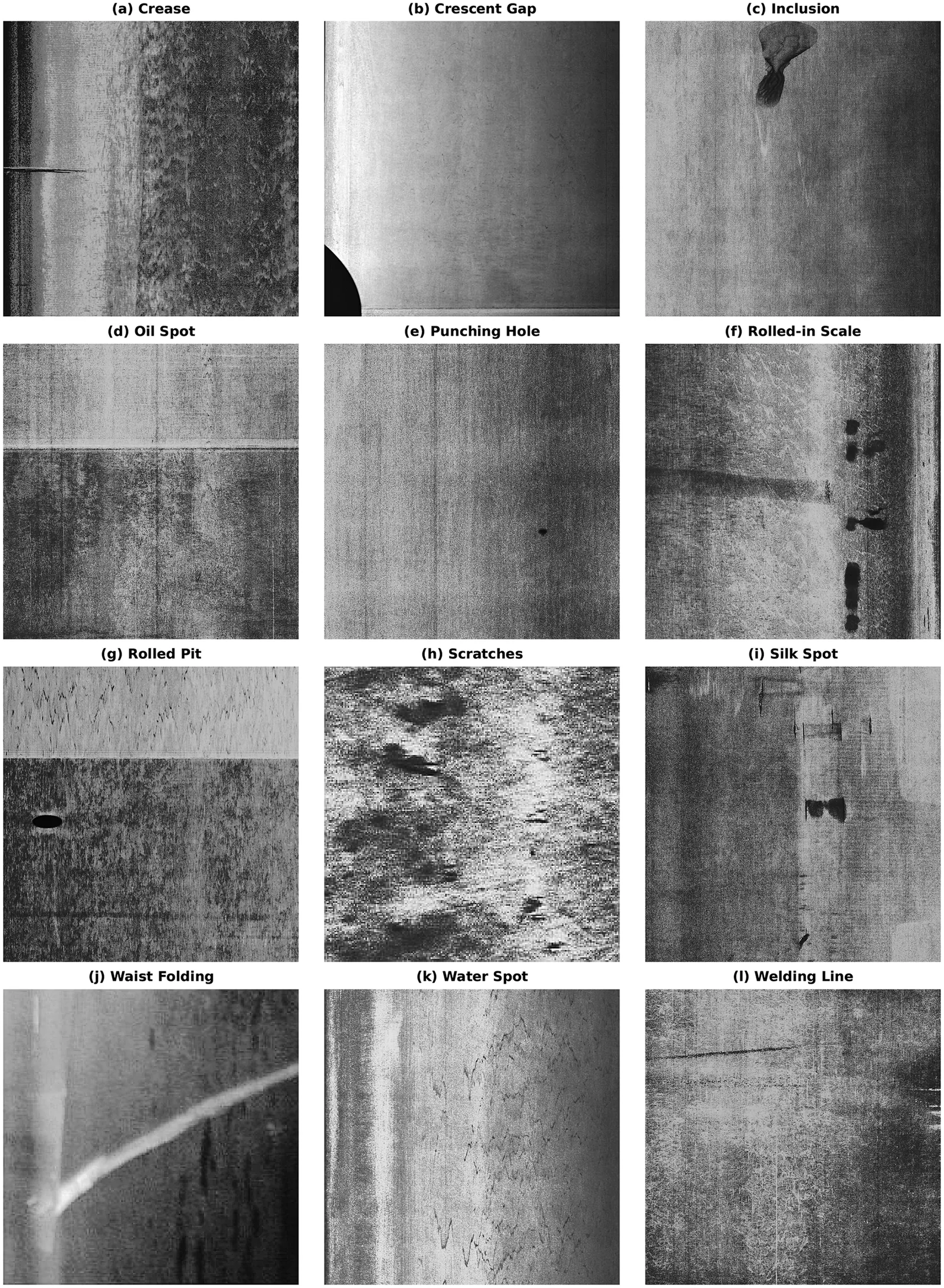
Examples from the metal surface defect dataset, showing all twelve categories. Images contrast-enhanced via histogram equalization for improved visibility. **(a)** Crease, **(b)** Crescent Gap, **(c)** Inclusion, **(d)** Oil Spot, **(e)** Punching Hole, **(f)** Rolled-in Scale, **(g)** Rolled Pit, **(h)** Scratches, **(i)** Silk Spot, **(j)** Waist Folding, **(k)** Water Spot, **(l)** Welding Line.

The metal surface defect dataset consists of 3,212 grayscale images of surface defects found in hot-rolled steel strips. The dataset is divided into twelve defect categories, namely crease, crescent gap, inclusion, oil spot, punching hole, rolled-in scale, rolled pit, scratches, silk spot, waist folding, water spot, and welding line (Figure 4). The images are generally sized at 200 × 200 pixels and challenges machine learning algorithm due to intra-class variation, inter-class overlap, and inconsistent lighting conditions across samples. Despite these challenges, the dataset is well-organized and cleanly structured, making it highly valuable for researchers and engineers for developing and testing automated defect detection systems for industrial and manufacturing applications.

**Figure 5:**
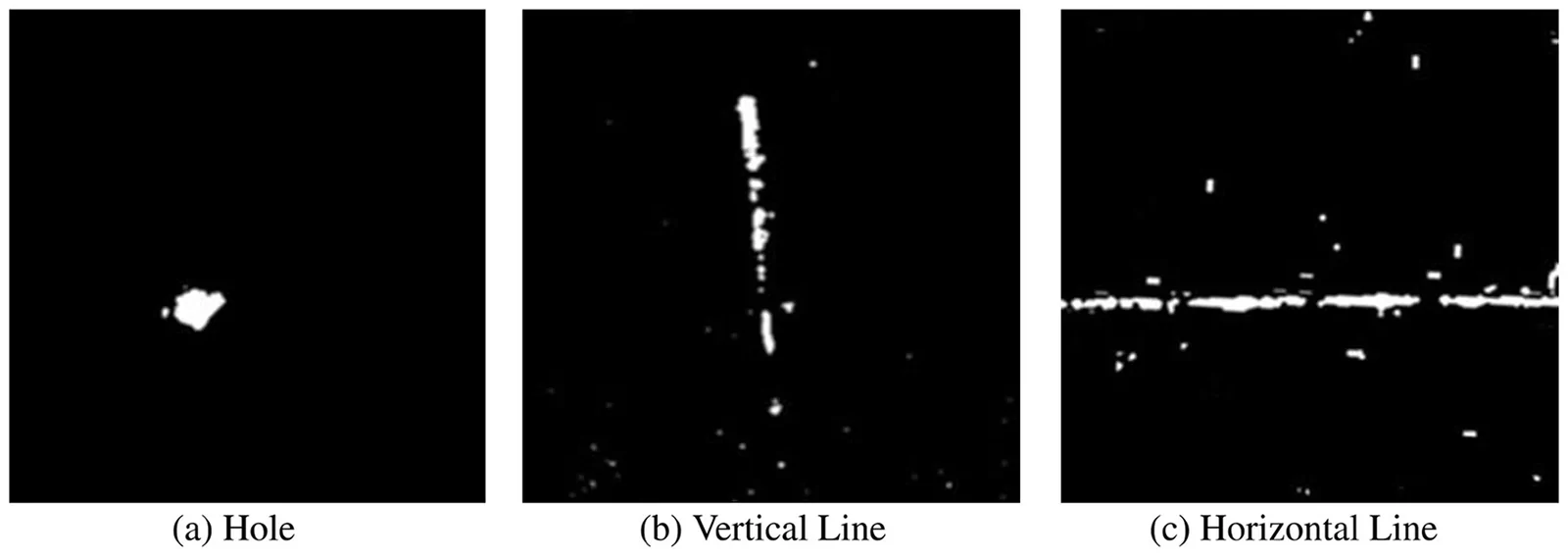
Examples from the fabric defect dataset. **(a)** Hole, **(b)** Vertical Line, **(c)** Horizontal Line.

The fabric defect dataset (Figure 5) consists images of fabrics which shows various flaws, including holes, horizontal lines, and vertical lines and then by categorizing data into classes to supports the development of machine learning models for classification and detection of defect. Which helps to improve quality control in textile manufacturing industries, the need of these type of datasets are crucial for optimal results and helps in automate manual inspections which are time consuming and have a risk of error. Leveraging the dataset helps in the development of more accurate automated systems, enhancing the overall efficiency and reliability of fabric assessment.

Table 1 presents the detailed distribution of images across the training, validation, and testing subsets for both the metal surface defect and fabric defect datasets. An 80:10:10 split ratio was adopted to ensure balanced model training and reliable evaluation. To prevent overfitting and enhance model generalizability, data augmentation techniques were applied to the training set, including random horizontal and vertical flipping, rotations of up to 15 degrees, and subtle color jittering. Furthermore, weight decay (*L*_2_ regularization) and early stopping mechanisms were actively employed during the training process. The Metal dataset contains 13 defect categories with a total of 6,226 images, while the Fabric dataset consists of three defect categories comprising 412 images. The distribution strategy ensures that each defect class is represented across all subsets, thereby reducing data bias and improving the generalization capability of the proposed HQCNN model. The larger number of samples in certain categories such as *lable* and *silk_spot* contributes to robust feature learning, whereas minority classes such as *rolled_pit* provide challenging scenarios for defect classification. Overall, the dataset partitioning establishes a consistent experimental setup for comparative performance analysis.

**Table 1:** Train, validation, and test distribution for metal and fabric defect datasets.

Dataset	Defect class	Total	Train (80%)	Validation (10%)	Test (10%)
Metal	crease	53	42	5	6
Metal	crescent_gap	226	181	23	22
Metal	inclusion	516	413	52	51
Metal	lable	3,014	2,411	301	302
Metal	oil_spot	204	163	20	21
Metal	punching_hole	219	175	22	22
Metal	rolled_in_scale	300	240	30	30
Metal	rolled_pit	31	25	3	3
Metal	scratches	300	240	30	30
Metal	silk_spot	651	521	65	65
Metal	waist_folding	150	120	15	15
Metal	water_spot	289	231	29	29
Metal	welding_line	273	218	27	28
Fabric	holes	184	147	18	19
Fabric	horizontal_lines	136	109	14	13
Fabric	vertical_lines	92	74	9	9
**Total**	—	**6638**	**5310**	**663**	**665**

Bold values indicate the performance of the proposed HQCNN model and its associated metrics.

The proposed HQCNN exhibits higher accuracy compared to classical models in the classification of defects in the fabric defects dataset. The HQCNN achieves an accuracy of 93.0 ± 1.8%, compared to the conventional convolutional neural network (CNN) at 86.0 ± 2.5% and the baseline K-nearest Neighbor (KNN) model at 75.0 ± 3.1%. This difference is further detailed in Table 2, which presents class-wise metrics where HQCNN shows higher precision, recall, and F1-scores for defects such as holes, horizontal lines, and vertical lines. These results advise that the addition of a six-qubit variational quantum circuit (VQC) enables robust feature representation, preparing it to capture complex defect patterns in fabrics. In contrast, KNN with its lower performance indicates its difficulty in capturing defect patterns in fabric because of its distance-based method, which may be less effective in capturing the data features required for this classification task.

**Table 2:** Comparison of algorithms for fabric defect dataset.

	Precision			Recall			F1-score			ROC-AUC		
Metric	HQCNN	CNN	KNN	HQCNN	CNN	KNN	HQCNN	CNN	KNN	HQCNN	CNN	KNN
Holes	**1.00**	0.91	0.75	**0.89**	0.84	0.88	**0.94**	0.87	0.81	**0.97**	0.90	0.82
Horizontal lines	**0.93**	0.79	0.56	**0.93**	0.90	0.71	**0.93**	0.84	0.62	**0.95**	0.87	0.76
Vertical lines	0.83	0.88	0.70	**1.00**	0.82	0.57	**0.91**	0.85	0.71	**0.94**	0.88	0.75
Accuracy	—	—	—	—	—	—	**0.93** ± 1.8	0.86 ± 2.5	0.75 ± 3.1	—	—	—
Macro Avg	**0.92**	0.86	0.75	**0.94**	0.85	0.72	**0.93**	0.85	0.72	**0.95**	0.88	0.78
Weighted Avg	**0.94**	0.86	0.78	**0.93**	0.86	0.75	**0.93**	0.86	0.74	**0.96**	0.89	0.77

Bold values indicate the performance of the proposed HQCNN model and its associated metrics.

### 4.2 Implementation details and reproducibility

All experiments were performed using the Python programming language with PyTorch and PennyLane libraries in GPU-powered cloud computing facilities. The suggested HQCNN combines an optimized ResNet-18 architecture for extracting classical features, a trainable quantum dynamic-depth model, and a classifier. All images were resized and normalized in accordance with a pre-defined consistent pipeline before their feature extraction.

Training of HQCNN models involved a batch size of 16 and an AdamW optimizer with an initial learning rate equal to 0.001. The number of training epochs amounted to 30 and 17 for Fabric Defects and Metal Surface Defects datasets correspondingly. For the sake of providing the same conditions for all experiments and model versions, all the optimization and preprocessing settings were kept the same for each fold and each version of the proposed models.

The quantum layer consists of the variational quantum circuit (VQC) with six qubits and a maximum of a four-layer depth. Every layer involves parameterized single-qubit rotation gates along with CNOT entangling gates arranged in a ring layout. Circuit execution has been performed using PennyLane's lightning.qubit state vector simulator. Backpropagation of gradients to both classical and quantum layers was performed using exact analytic gradients computed with the parameter-shift rule.

The classification results on the extremely unbalanced defect datasets were carefully analyzed based on Accuracy, macro F1-score, ROC-AUC, Matthews correlation coefficient (MCC), and Cohen's kappa. In order to achieve high reproducibility of the results, it is important to note that all models are trained under the same initialization, training procedure, and data preprocessing process. All mentioned metrics above are calculated as means of performance and its standard deviation for each cross-validation fold.

It is crucial to emphasize that the difference between the proposed approach and classical approaches to designing quantum classifiers lies in the usage of a trainable depth control layer based on the Gumbel-Softmax algorithm within HQCNN, while other methods are based on the use of predefined depth for all samples. Thus, the architecture allows for efficient resource allocation without losing classification performance.

### 4.3 Results and discussion

**Figure 6:**
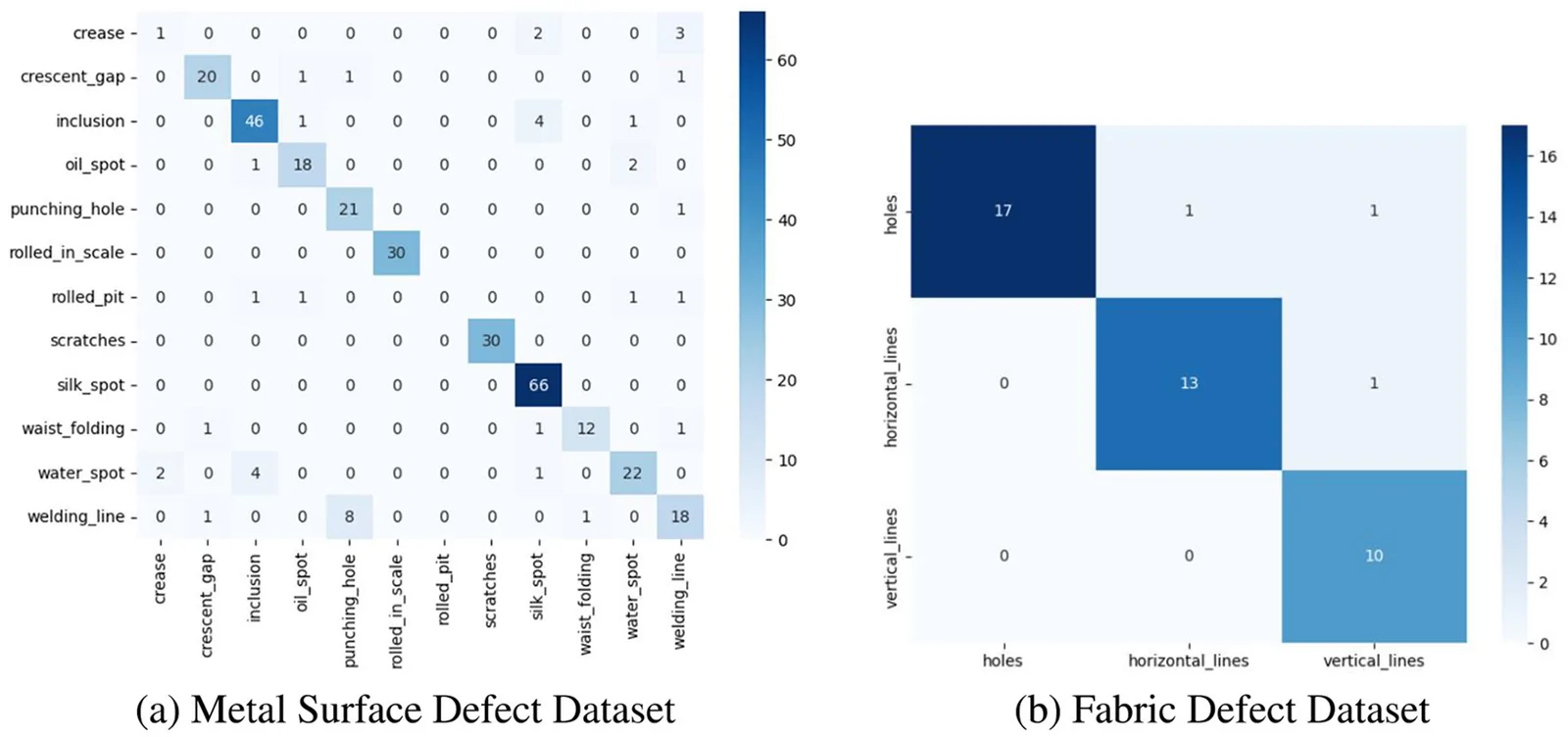
Confusion matrices for the metal and fabric surface defect dataset. **(a)** Metal Surface Defect Dataset, **(b)** Fabric Defect Dataset.

A variety of metrics is used to evaluate the performance of classification of models, which provides more insight than basic accuracy. These metrics clarify the different errors that occur and how well the model distinguishes among various classes. Some of the important metrics are precision, recall, F1-score, ROC-AUC, and accuracy. A detailed summary of these performance metrics and their corresponding equations is provided in Table 3. These metrics can be calculated from the confusion matrix. A confusion matrix summarizes the performance of a classification model by displaying the number of true positive (TP), true negative (TN), false positive (FP), and false negative (FN) predictions. We applied the HQCNN to the metal surface defect dataset and the fabric defect dataset. Relative to other models, the results indicated that the HQCNN achieved effective multi-class defect classification in these experiments. The results in the Metal Surface Defect dataset are illustrated by the confusion matrix (Figure 6a), which characterizes 12 classes of defects. The model achieved an overall accuracy of 88.1 ± 2.4% over 326 test samples. On a per-class basis, categories such as Inclusion (precision = 0.88, recall = 0.88, F1 = 0.88), scratches (1.00, 1.00, 1.00), silk spot (0.89, 1.00, 0.94), and rolled-in scale (1.00, 1.00, 1.00) showed high scores. While categories such as rolled pit introduced challenges, weighted-average scores (precision = 0.87, recall = 0.87, F1 = 0.87, ROC-AUC = 0.92) indicated consistent classification across most defect types. These results indicate that HQCNN can differentiate between categories of defects on steel surfaces, demonstrating its practical value for industrial applications. In addition, the performance of this model on unbalanced datasets is supported by a Matthew's correlation coefficient (MCC) of 0.887 and a Cohen's kappa of 0.880, which indicates a strong agreement between predictions and true labels. High accuracy was also obtained when the model was tested on a three-class fabric defect dataset, achieving an overall accuracy of 93.0 ± 1.8%. The confusion matrix provided a detailed performance report (Figure 6b). For the Vertical Lines class, a recall of 1.00 was achieved by the model, which means that all instances of these defects were identified, with a precision of 0.83. In contrast, the Holes class achieved a precision of 1.00 with a recall of 0.89. The horizontal lines class demonstrated balanced performance, achieving both precision and recall of roughly 0.93. These results indicate effective classification ability, further emphasized by a Matthews correlation coefficient (MCC) of 0.9120 and a Cohen's kappa of 0.9282.

**Table 3:** Key metrics, performance metrics, and equations.

Metric	Description	Equation
True positives (TP)	The number of actual positive instances that the model accurately classifies as positive.	TP = Correctly predicted positive instances
False positives (FP)	The cases where the model misclassifies negative instances as positive.	FP = Total predicted positives − TP
False negatives (FN)	The number of actual positive instances the model incorrectly classifies as negative.	FN = Total actual positives − TP
True negatives (TN)	The number of true negative instances the model correctly labels as negative.	TN = Total samples−(TP+FP+FN)
Precision	The percentage of true positives among all positive predictions made.	$\text{Precision}=\frac{\text{TP}}{\text{TP}+\text{FP}}$
Recall	The proportion of correct positive instances that are detected.	$\text{Recall}=\frac{\text{TP}}{\text{TP}+\text{FN}}$
F1-score	The harmonic mean of precision and recall.	$\text{F1-Score}=2\cdot \frac{\text{Precision}\cdot \text{Recall}}{\text{Precision}+\text{Recall}}$
Accuracy	The overall proportion of correct predictions.	$\text{Accuracy}=\frac{\text{TP}+\text{TN}}{\text{TP}+\text{TN}+\text{FP}+\text{FN}}$
ROC-AUC	Measures the two-dimensional area underneath the Receiver Operating Characteristic curve.	Evaluated integrally across varying classification thresholds.
MCC	A balanced measure that is robust to class imbalance, producing a high score only if the classifier performs well on both positive and negative classes.	$\text{MCC}=\frac{\text{TP}\times \text{TN}-\text{FP}\times \text{FN}}{\sqrt{\left(\text{TP}+\text{FP}\right)\left(\text{TP}+\text{FN}\right)\left(\text{TN}+\text{FP}\right)\left(\text{TN}+\text{FN}\right)}}$
Cohen's kappa	A statistic that measures the agreement between the model's predictions and the ground truth, while accounting for the possibility of the agreement occurring by chance.	$\kappa =\frac{p_{o}-p_{e}}{1-p_{e}}$

The outcome of the experiments indicated that the HQCNN is an effective model for multi-class defect classification in these settings. A summary of the main results of the two key experiments is presented in Table 4. Table 5 presents a comparison between the proposed HQCNN and existing hybrid models discussed in recent literature. The dynamic-depth architecture maintains high classification accuracy while facilitating data-adaptive quantum resource utilization.

**Table 4:** Summary of main experimental results for the HQCNN model.

Dataset	Accuracy (%)	Precision	Recall	F1-score	ROC-AUC
Fabric	93.0 ± 1.8	0.94 ± 0.02	0.93 ± 0.02	0.93 ± 0.02	0.96 ± 0.01
Steel	88.1 ± 2.4	0.87 ± 0.03	0.87 ± 0.02	0.87 ± 0.02	0.92 ± 0.02

**Table 5:** Comparison of HQCNN with existing hybrid quantum-classical models.

Model and reference	Application area	Classical backbone	Quantum architecture	Reported accuracy
HQCNN (Khurelsukh, 2022)	Facial emotion recognition	Classical CNN	Hybrid QCNN (re-uploading)	70.25%
QCNN (Reejisha and Mohan, 2023)	Fashion image classification	Dense layers	Static QCNN	86.0%
**Proposed HQCNN**	**Industrial defect detection**	**ResNet-18**	**Dynamic VQC (Gumbel-Softmax)**	**88.1%–93.0%**

Bold values indicate the performance of the proposed HQCNN model and its associated metrics.

From these results, it is evident that Dynamic HQCNN manages to meet the balance between power requirements due to complexity in images and efficiency to save resources, outperforming the fixed-depth strategy with a superior F1-score and accuracy compared to the classical baseline method, as seen in Table 6. In order to provide an explicit rationale for the use of the quantum variational circuit against purely classical approaches, a structural bottlenecking and parameter-matching ablation study was conducted. The performance of ResNet-18 when forced to reduce its features into a 6-dimensional classical bottleneck similar to the six-qubit quantum circuit decreases, yielding an accuracy of 76.50 ± 3.5% and a macro F1-score of 0.7598 ± 0.032. This indicates a significant loss of information regarding the minority classes. Moreover, replacing the six-qubit VQC with a classical MLP with a matching number of parameters yields a lower accuracy of 78.22 ± 2.8%, and its Macro F1-score decreases significantly to 0.7944 ± 0.025 compared to the 0.8700 ± 0.022 F1-score achieved by the six-qubit model. The primary distinction here is that while classical densely structured layers and structural bottlenecking overfit to the majority classes to maintain good accuracy, the quantum approach successfully learns highly discriminative features for the minority defects. In addition to this, to confirm the effectiveness of the Gumbel-Softmax depth controller, a comprehensive evaluation of fixed-depth quantum circuits was performed, with depths ranging from *d* = 1 to *d* = 4. Based on the experimental findings, there appears to be an inherent limitation in fixed-depth quantum circuits: requiring that all input samples go through one circuit depth level limits the maximum value of the macro F1-score to about 0.8167. With a shallow depth of *d* = 1, the neural network does not have sufficient parameterization capability to classify complicated and overlapping defect types, whereas deeper quantum circuits (*d* = 3, *d* = 4), although slightly inferior in performance, may be subjected to overfitting and trainability problems. As for the proposed approach, the dynamic HQCNN dynamically adjusts the circuit depth according to the level of difficulty in processing input images. Therefore, with an average depth of 2.4, the proposed network circumvents the drawbacks of static allocation by obtaining the highest macro F1-score of 0.8700 ± 0.022.

**Table 6:** Comprehensive ablation study on the metal surface defect dataset.

Model architecture	Bottleneck/VQC params	Accuracy (%) ±SD	Macro F1 ±SD	Avg depth
Classical baselines				
Pure classical ResNet-18	6,144 (dense FC)	86.90 ± 2.2	0.8300 ± 0.021	N/A
Classical six-dim bottleneck	72	76.50 ± 3.5	0.7598 ± 0.032	N/A
Classical matched MLP	49	78.22 ± 2.8	0.7944 ± 0.025	N/A
Quantum baselines				
Fixed depth VQC (*d* = 1)	12	78.16 ± 3.0	0.7831 ± 0.029	1.0
Fixed depth VQC (*d* = 2)	24	79.71 ± 2.6	0.8167 ± 0.024	2.0
Fixed depth VQC (*d* = 3)	36	78.40 ± 2.9	0.7951 ± 0.028	3.0
Fixed depth VQC (*d* = 4)	48	77.50 ± 3.4	0.8000 ± 0.031	4.0
Dynamic controller ablations				
Dynamic (random routing)	Up to 48	77.24 ± 3.2	0.7752 ± 0.033	2.5
Dynamic (no penalty, λ = 0)	Up to 48	79.47 ± 2.7	0.8005 ± 0.026	2.4
**Dynamic HQCNN**	**Up to 48**	**88.10** **±2.4**	**0.8700** **±0.022**	**2.4**

Bold values indicate the performance of the proposed HQCNN model and its associated metrics.

Finally, to rigorously evaluate the structural necessity of the Gumbel-Softmax depth controller and its objective function, two dynamic control conditions were introduced (Table 6). First, replacing the learned routing with random depth assignment caused the macro F1-score to drop to 0.7752. This explicitly confirms that the HQCNN does not merely benefit from varied quantum noise, but actively learns an intelligent mapping between specific defect morphologies and optimal quantum circuit depths. Second, removing the expected depth penalty (λ = 0) from the loss function resulted in a macro F1-score of just 0.8005. While the unpenalized model maintained a similar average depth, the absence of the penalty regularizer allowed the controller to make indecisive, unconstrained routing choices. The full Dynamic HQCNN, driven by the penalized loss function, achieves a macro F1-score of 0.8700, indicating that the penalty acts as an effective architectural regularizer that forces the network to specialize its quantum pathways for complex, minority-class defects.

**Figure 7:**
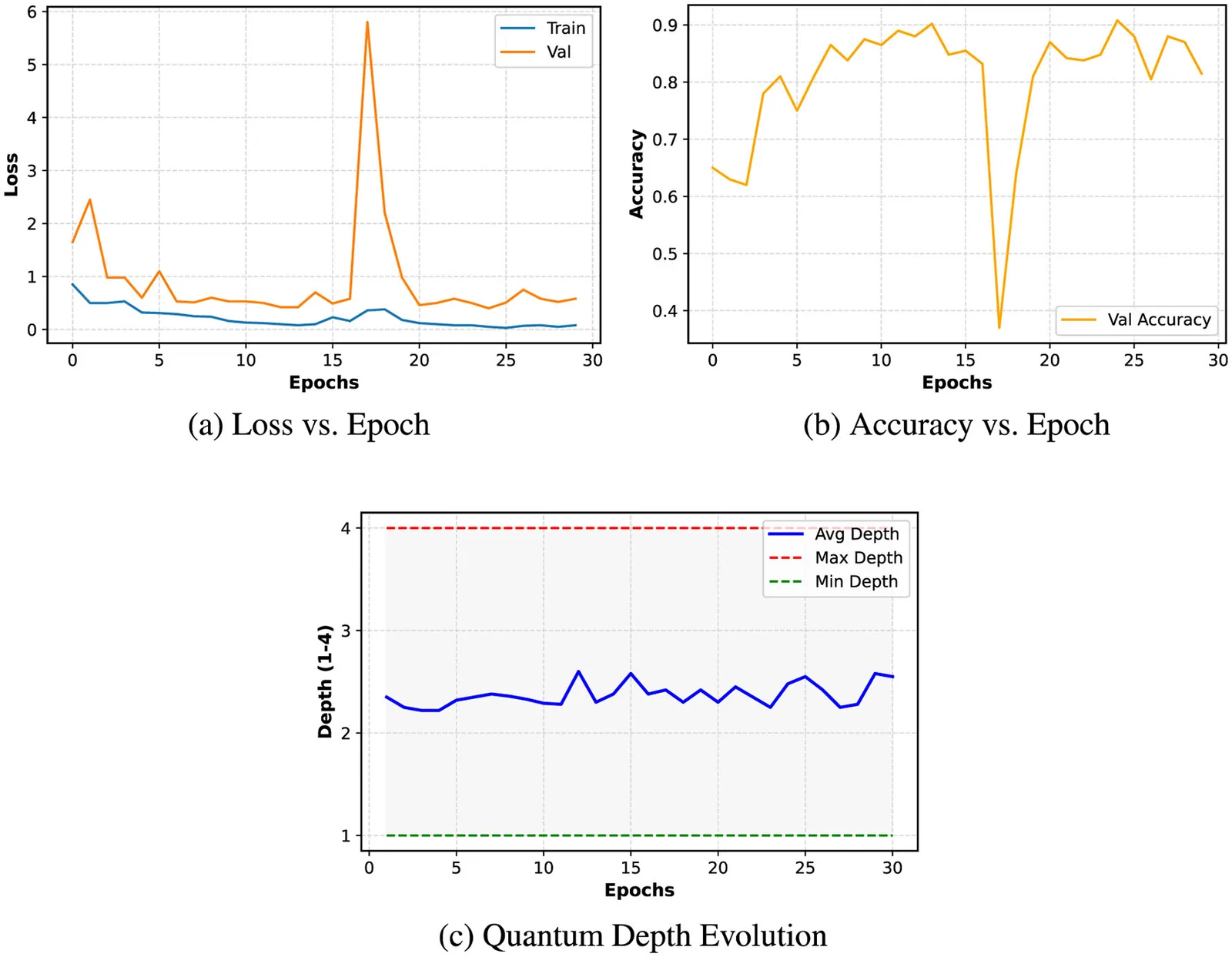
Training metrics for the fabric defect dataset: **(a)** continuous training and validation loss reduction over 30 epochs, **(b)** the corresponding validation and training accuracy improvement, and **(c)** the dynamic quantum depth adjustments made by the controller during the training phase, illustrating the average depth convergence.

The HQCNN was evaluated on the fabric defects dataset and the 12-class metal surface defect dataset, which showed consistent training behavior across experiments. In the Fabric Defect dataset, the model achieved a test accuracy of 93.0%. The training history (Figure 7b) shows that the validation accuracy peaked at approximately 91.0%; simultaneously, the training loss (Figure 7a) converged to near 0.05 while the validation loss stabilized around 0.55 after a short spike in epoch 17. Throughout the training, the dynamic depth controller maintained an active search space, reflected by an average depth oscillating around 2.4 (Figure 7c).

**Figure 8:**
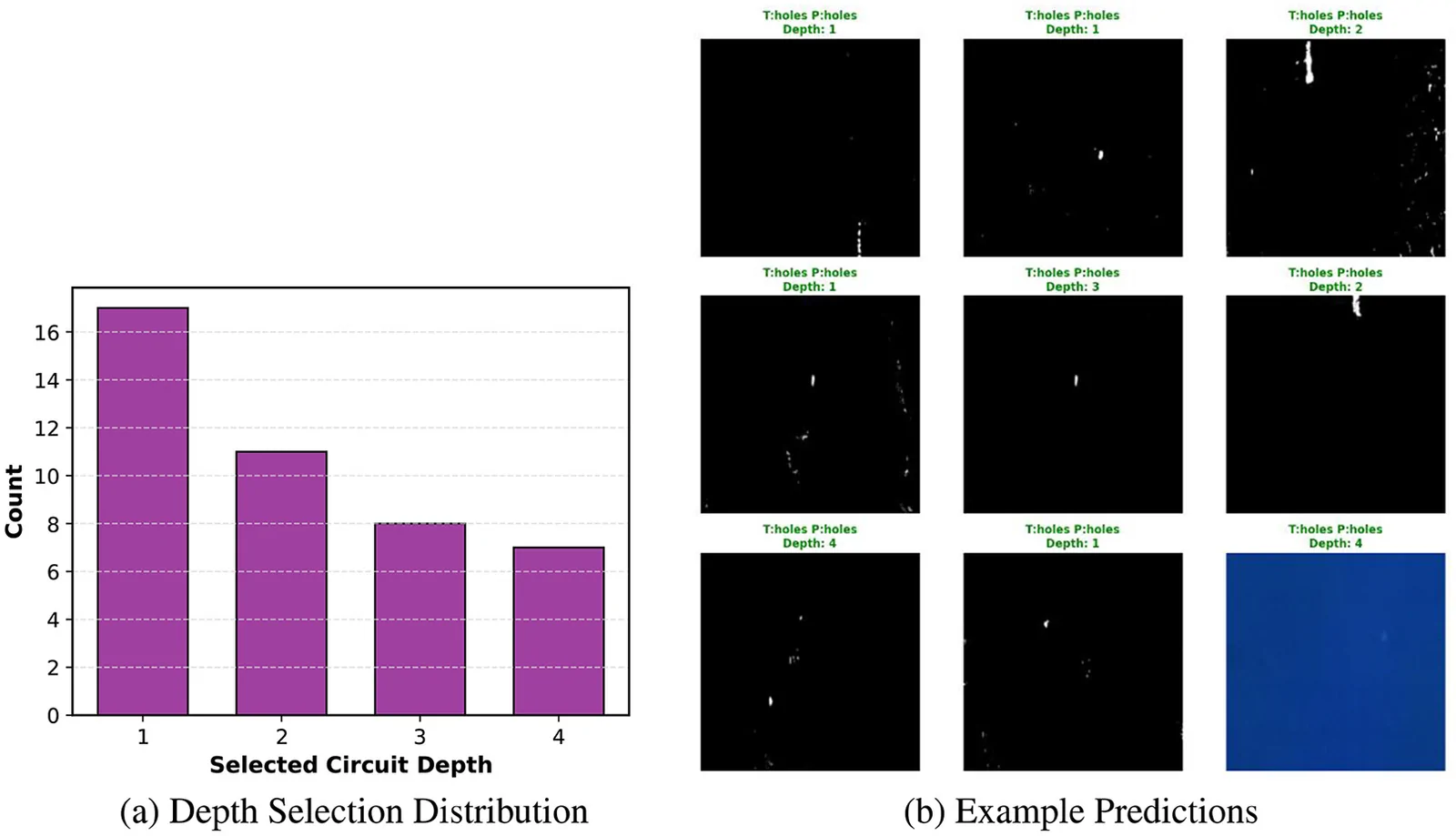
Analysis of the fabric defect dataset test set: **(a)** A histogram detailing the distribution of circuit depths selected by the controller, demonstrating efficient resource routing. **(b)** Accurate prediction examples showcasing the model's adaptive depth assignment for various defect morphologies.

As confirmed in the depth selection distribution (Figure 8a), which illustrates the controller's behavior on the test data, the model selected the shallowest circuits (depth 1) for the highest number of test samples, having a total of 17 instances, followed by depth 2 having 11 instances, while reserving deeper configurations of depth 3 (eight instances) and a max depth of 4 (seven instances) for deep feature extraction. The controller maintained an effective balance between quantum search space exploration and resource utilization. Further validation of this strategy is provided by the visual analysis of predictions in Figure 8b, where defects such as holes were identified across varying circuit complexities of depth 1, 2, and 4, indicating that HQCNN can extract discriminative features without uniformly maximizing the quantum cost.

**Figure 9:**
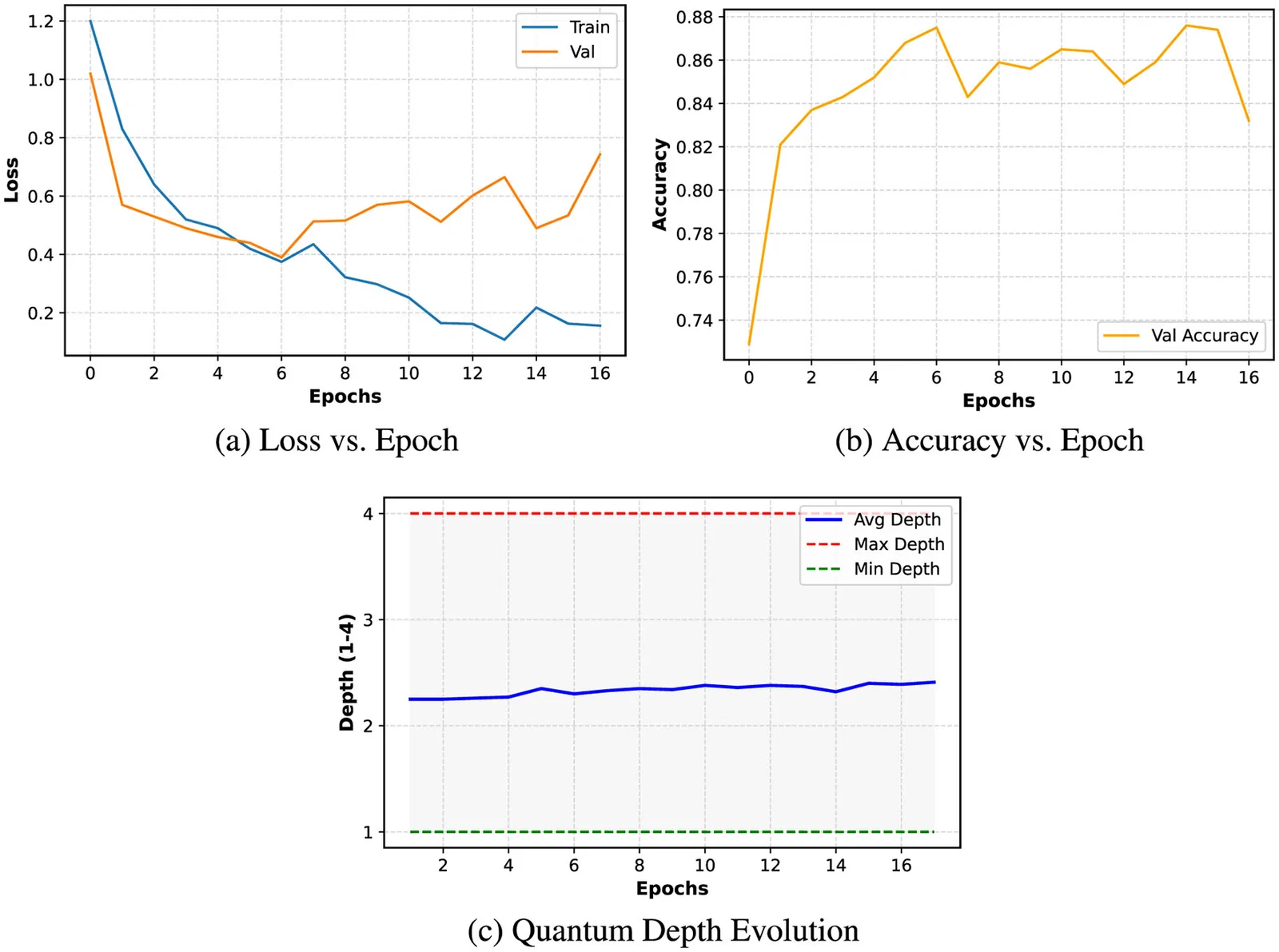
Training metrics for the metal surface defect dataset: **(a)** training and validation loss decay, **(b)** progressive accuracy improvement throughout epochs, and **(c)** the dynamic average depth adjustments stabilizing near 2.4.

**Figure 10:**
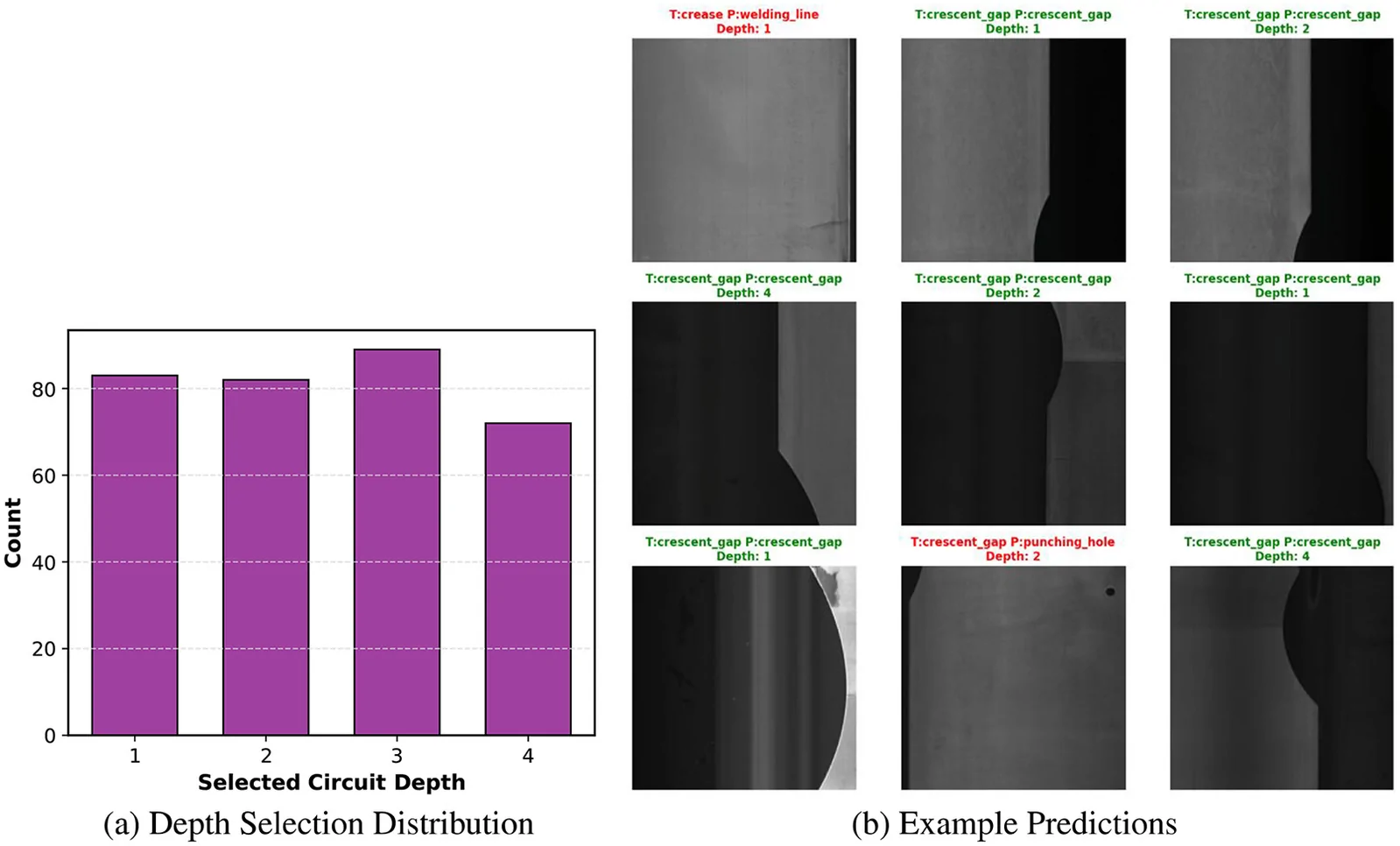
Analysis of the metal surface defect dataset test set: **(a)** Depth selection distribution illustrating preference for moderate circuit complexities. **(b)** Accurate multi-class prediction examples alongside their dynamically assigned circuit depths.

To rigorously evaluate the model, the metal surface defect dataset was used, which represents a 12-class classification task, and the model achieved a test accuracy of 88.1%, indicating the adaptive nature of the model to maintain the prediction accuracy of the defect on different materials. In the training phase, the validation accuracy improved in the early epochs, reaching 85.2% by epoch 4, and then stabilized in the range of 84%–88% across training (Figure 9b). This training history is also demonstrated by the loss curves (Figure 9a), where the training loss narrowed down to approximately 0.16 by the final epochs, while the validation loss stabilized around 0.5–0.6. The controller maintained a consistent active search space with an average circuit depth fluctuating between 2.3 and 2.4 throughout the training process (Figure 9c). This is further confirmed by the depth selection distribution in Figure 10a, which shows how the resources are allocated. The most frequently utilized depth by the model is Depth 3 with 89 instances, followed by depth 1 having 83 instances and depth 2 with 82 instances, while the deepest configuration, depth 4, was used least often, having 72 instances. These results highlight the adaptive nature of the depth mechanism of HQCNN for this problem to use resources effectively while not compromising on accurate prediction, and the mixed-depth strategy provided a balance between representational power and robustness. The predictions in Figure 10b further confirm the ability of the model to generalize across diverse defect categories, demonstrating that precision can be achieved by dynamically adapting quantum resources, correctly classifying *crescent_gap* at depths 1, 2, and 4.

### 4.4 Industrial deployment and study limitations

While the proposed HQCNN demonstrates potential for automated defect detection, transitioning this hybrid framework into a real-world industrial deployment pipeline presents distinct practical challenges. The current empirical evaluations rely on state-vector simulators (PennyLane), which are highly optimized but fundamentally abstract away the quantum noise, gate infidelities, and decoherence errors inherent in physical NISQ hardware. In a high-throughput manufacturing environment, such as real-time hot-rolled steel inspection, the latency introduced by quantum-classical data encoding (e.g., angle embedding) and cloud-based QPU access could bottleneck the system. Deploying this model in an industrial setting would require edge-compatible quantum simulators or fault-tolerant on-premise QPUs to satisfy stringent inference latency requirements. Furthermore, the mean training time per epoch was recorded as 112.4 ± 5.6 s, inference time per sample was 14.2 ± 1.1 ms, and peak memory usage reached 4.8 GB. This indicates that although the classification head is parametrically efficient, state-vector simulation still incurs a non-trivial computational overhead on classical hardware.

Several limitations of this study must also be acknowledged. First, the reliance on noise-free simulators restricts the current understanding of how environmental quantum noise might impact the routing decisions made by the Gumbel-Softmax dynamic depth controller. Second, although the HQCNN generalizes well across the 12-class metal and three-class Fabric datasets, the scalability of the six-qubit architecture to accommodate considerably higher-resolution imagery or an expanded set of complex defect morphologies remains to be empirically validated. Increasing the qubit count to enrich the feature space would exponentially increase the computational overhead of classical simulation, constraining extensive prototyping. Finally, future work must focus on executing these dynamic models directly on physical quantum hardware and developing noise-resilient routing mechanisms that account for the physical layout and error rates of specific quantum processors.

## 5 Conclusion

This work presented a Hybrid Quantum Classical Neural Network (HQCNN) for automated defect detection in materials such as fabrics and steel. By integrating a fine-tuned ResNet-18 backbone with a dynamic variational quantum circuit (VQC), the proposed framework gives an edge in compatible strengths of classical deep learning and quantum feature extraction. Experimental results indicated classification accuracy across the two benchmark datasets of fabric and steel having three and 12 classes for classification respectively used in this study, achieving better performance in the fabric defects dataset and sturdy results on the challenging metal surface defect dataset. These findings focus on the ability of HQCNN to extract unique features with optimal quantum resource usage, suggesting the feasibility of such models for deployment under limited computational resources. The adaptive depth controller not only ensures proper use of quantum resources but also contributes to model durability. These characteristics position HQCNN as a promising approach for a scalable, intelligent, and resource-aware quality control mechanism in modern manufacturing. Future research should aim to expand datasets to include a wider range of defect categories and real-world industrial variations, optimize quantum circuit architectures for scalability to larger qubit systems, and comprehensively evaluate deployment on actual quantum hardware. Addressing these limitations will support the building of more reliable, cost-effective, and quantum-enhanced automated defect detection systems that can refine the standards of quality assurance in industry.

## Data Availability

Publicly available datasets were analyzed in this study. This data can be found here: https://ieee-dataport.org/documents/fabricdefect.
